# A Qualitative Study of the Oxidation Resistance of PBF-LB/M High-Ta Ni-Based Superalloys with Hf Additions

**DOI:** 10.3390/ma19081482

**Published:** 2026-04-08

**Authors:** Kai Dörries, Joachim Rösler

**Affiliations:** Institut für Werkstoffe, Technische Universität Braunschweig, Hans-Sommer-Straße 5, 38106 Braunschweig, Germany; j.roesler@tu-braunschweig.de

**Keywords:** Ni-based superalloy, oxidation, additive manufacturing, alloy development

## Abstract

Recent studies have shown that a new family of Ni-based superalloys with high Ta and high Hf contents exhibits a promising property profile and may be able to fill the gap between creep-resistant alloys and those processable by PBF-LB/M. The effect of simultaneously high Ta and Hf contents on oxidation resistance has not yet been investigated and is addressed qualitatively in this study. Isothermal oxidation tests were conducted in air at 950 °C for 100 h, 300 h, and 500 h. After cooling, the weight change and cross-sections of the specimens were examined. The study shows that the Hf-free alloy exhibits severe spallation of the Al-oxide and Cr-/Ni-oxide layer. The Hf-containing alloys exhibit improved oxide layer adhesion and a promoted formation of a continuous Al-oxide layer, which is attributed to the early formation of Hf-oxide particles. Furthermore, the addition of Hf influences the morphology of internally oxidized Al, which grows preferentially parallel to the surface rather than perpendicular to it. This behavior leads to effective protection of the alloys by an Al-oxide layer, either external or internal, which is remarkable considering the moderate Al content of only 3 wt.%.

## 1. Introduction

Ni-based superalloys are known for their outstanding high-temperature resistance and therefore essential for the hot gas path of gas turbines that are used in the aerospace or energy sector. Additive manufacturing technologies such as powder bed fusion by laser beam of metals (PBF-LB/M) are already used to manufacture turbine components, because they allow more complex features that increase the efficiency of gas turbines. However, the number of well-suited Ni-based superalloys for PBF-LB/M is limited to materials with low volume fractions of the γ′-phase and low amounts of minor elements (like B or Zr), because they cause strain-age cracking and hot cracking [1]. Unfortunately, those are needed for creep resistance at high temperatures, which limits the service temperature of current PBF-LB/M processed gas turbine components. Therefore, new alloys that combine processability and creep resistance are needed.

To overcome these limitations, a new generation of alloys was developed specifically for PBF-LB/M processing. All three of those alloys contain 9% Ta and 3% Al, which leads to a high positive γ/γ′-lattice mismatch. The idea behind this measure is to increase the strength of the alloy without increasing the Al content too much, because a high Al content leads to strain-age cracking (SAC) during subsequent heat treatments [1,2,3]. The addition of ≥1.3% Hf to the alloy system leads to a significant increase in stress-rupture lifetime and elongation at fracture. This is believed to be caused by the formation of alternating coarse γ′-particles and γ-seams at grain boundaries [1]. Unfortunately, it also leads to the occurrence of either hot cracks (at 1.3% Hf) or SAC (at 1.9% Hf) [1].

However, creep resistance and processability are not the only requirements that need to be fulfilled. Alloys designed for high-temperature applications not only have to exhibit a high creep resistance, but also a good oxidation resistance. Most of the parts that are exposed to high temperatures (>800 °C) are protected by Cr- and/or Al-rich coatings, but those can be damaged during service. Additionally, internal surfaces like the cooling channels of turbine blades remain unprotected, but are still exposed to a high temperature that leads to oxidation. Thus, the base materials must also be resistant to oxidation to ensure a long service lifetime.

The oxidation resistance of these newly developed alloys has not been investigated yet, and there are no studies available on the oxidation resistance of high Ta containing Ni-based superalloys with high Hf contents. Therefore, an initial isothermal oxidation test was conducted on three candidate alloys during the development of a new Ni-based superalloy for additive manufacturing to investigate how variations in chemical composition influence the oxidation resistance of the alloys. In this study, the isothermal oxidation resistance of three variations of the SAM (superalloy for additive manufacturing) family with no hafnium addition, 1.3 wt.% Hf, and 1.9 wt.% Hf is examined. The samples were produced by PBF-LB/M and tested for 100 h, 300 h, and 500 h at 950 °C with subsequent air cooling.

In general, Ni-based superalloys are protected by a Cr_2_O_3_ and/or Al_2_O_3_ layer and can be classified into three types of alloys. Type I alloys form an outer NiO scale above a Cr_2_O_3_ or Al_2_O_3_ scale. Type II alloys form an outer Cr_2_O_3_ layer with internally oxidized aluminum particles and type III alloys form a continuous outer Al_2_O_3_ layer [4]. Alloys that form an outer Cr_2_O_3_ layer and a continuous Al_2_O_3_ layer underneath are classified as type III alloys as well [4,5,6].

The oxidation type depends strongly on the Cr and Al content, but other elements also affect the oxidation resistance. The baseline composition of the three investigated Ni-based superalloys in this study stands out with its high Ta content of 9 wt.% and the absence of Ti. The alloys are designed specifically for processing by PBF-LB/M, and the high Ta content is one measure to achieve a low SAC susceptibility with a simultaneously high creep resistance [1,2,3]. However, Ta also affects the oxidation resistance by slowing down the diffusion of other elements [5,7], such as Al or O, or forming oxide layers such as Ta_2_O_5_ [8,9], NiTa_2_O_6_ [5], or CrTaO_4_ [10]. Whether the effect of Ta additions is beneficial or detrimental seemingly depends on the general composition of the alloy, the temperature, and the amount of Ta.

It appears to be a common thread that low amounts of Ta of around 1 at.% (~3 wt.%) improves the oxidation resistance by forming a protective NiTa_2_O_6_ layer [11] or by promoting the formation of a protective Al_2_O_3_ layer [9]. However, there are conflicting reports about the effect of high amounts of Ta. Chang et al. [8] report that a high Ta/Cr ratio with a Ta content above 6 wt.% leads to a competitive growth of Ta_2_O_5_ and Cr_2_O_3_ that destroys the compactness of the oxide layer and thereby deteriorates the oxidation resistance. Similarly, the study of Yang [9] shows that 3 at.% (~9 wt.%) leads to the formation of Ta_2_O_5_ that reduces the stability of the protective Al_2_O_3_ layer. Barrett et al. [12] conducted an extensive study, in which they investigated the effect of many alloying elements on the oxidation resistance. They found that alloys with a high Ta content of around 3 at.% display a better oxidation resistance, but the mechanisms remained unclear. A study of Park et al. [7] gives some insight on why the threshold of the reversal of Ta’s effect on oxidation resistance differs from study to study. They found that a high Ta content is beneficial at high temperatures such as 1000 °C, but not at 850 °C. Additionally, its effect is beneficial if the amount of Al is as low as 4 wt.%. This is linked to the influence of Ta on the diffusivity of O and Al, which depends on the temperature and Al content.

The Ni-based superalloys investigated in this study include two Hf-containing alloys and one Hf-free alloy. The addition of Hf to this new Ni-based superalloy increases the stress-rupture lifetime significantly, stabilizes the MC-carbides, and affects the susceptibility to strain-age cracking (SAC) and hot cracking [1]. However, Hf is also known to affect the oxidation resistance of Ni-based superalloys. It is generally agreed that small additions of hafnium, e.g., 0.08–0.4 wt.%, are beneficial to the oxidation resistance [13,14,15], but an overdoping of Hf will decrease the oxidation resistance compared to that of small additions [14,16]. The addition of Hf increases the scale adhesion [13,14,15,17,18,19], which is especially beneficial for Al_2_O_3_-forming alloys that are susceptible to spallation [20]. The increased adhesion is linked to a couple of reasons.

One of them is that Hf binds sulfur and prohibits it from diffusing to the oxide/metal interface, where it would otherwise decrease the adhesion [14,20,21]. Additionally, Hf forms HfO_2_ particles instead of a continuous oxide layer, which are called pegs, that grow perpendicular to the surface. These HfO_2_ particles are nucleation sites for Al_2_O_3_, which lead to a stronger adhesion between the Al_2_O_3_ layer and the metal substrate [17,18,19,21]. Hf is also reported to decrease the growth rate of the Cr_2_O_3_ or Al_2_O_3_ layer [13,15,17,22]. In the Al_2_O_3_ layer, it is believed to segregate to the grain boundary and reduce the outwards diffusion of Al^3+^ cations [15,22]. The reduced thickness of the Al_2_O_3_ improves the adhesion by reducing strain energy during cooling [22].

Similar to Ta, Hf needs to be added with care, since an overdoping can cause the deterioration of the oxidation resistance. It is reported that excessive addition of Hf leads to the formation of Hf-rich instead of Ta- and Ti-rich carbides, which can negatively affect the oxidation resistance [14]. In another study, the oxidation of Hf rich carbides at the surface led to the formation of Al_2_O_3_ around those carbides, which resulted in an insufficient amount of Al to form a continuous Al_2_O_3_ layer above those carbides at the surface, ultimately deteriorating the oxidation resistance of the alloy [16]. However, there are also reports where a high Hf content above 2 wt.% results in a better oxidation resistance [17,18].

## 2. Materials and Methods

Three new Ni-based superalloy powders were fabricated via vacuum induction melting followed by inert gas atomization at VDM Metals International GmbH (Werdohl, Germany). Argon served both as the atomization medium and protective atmosphere. Subsequent air classification and sieving yielded particles with sizes ranging from 15 µm to 53 µm. The chemical composition of the as-atomized powders was determined using wet chemical analysis and is listed in Table 1.

Before processing, the build chamber of the SLM 125 HL system (Nikon SLM Solutions AG, Lübeck, Germany) was purged with argon until the oxygen level dropped below 0.05 vol.%. The samples were then fabricated using a layer thickness of 40 µm, with the scan vector rotated by 67° between successive layers. All samples were processed with the same parameter set that results in a volumetric energy density of 45 J/mm^3^.

**Figure 1:**
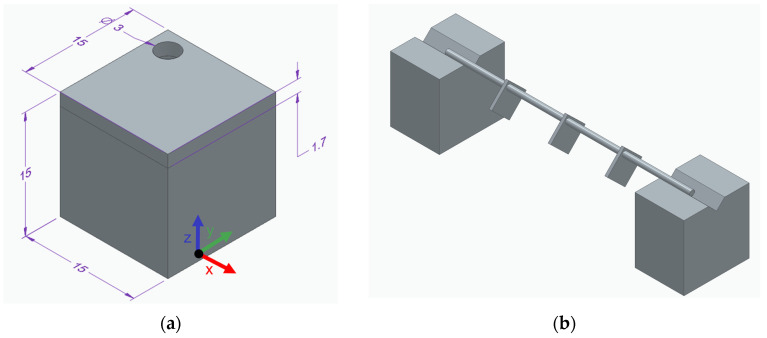
(**a**) Dimensions (in mm) of the printed cubes and the oxidation specimens extracted from them. The build direction corresponds to the z-direction, from bottom to top. (**b**) Experimental setup showing the oxidation specimens suspended on a wire inside the furnace during oxidation testing.

A cube with an edge length of 15 mm containing a hole with a 3 mm diameter was processed (see Figure 1a). The cubes were heat-treated in a vacuum furnace with a heating rate of 15 K/min. SAM-Zr was solution annealed at 1250 °C, SAM-1.3Hf at 1230 °C, and SAM-1.9Hf at 1180 °C. The solution annealing temperatures were chosen according to the incipient melting temperatures of the alloys, which are decreased by the addition of Hf. However, the respective solution annealing temperature leads to a complete homogenization in all three alloys, because it is higher than the respective γ′-solvus and lower than the respective solidus temperature. Therefore, the different temperatures do not affect the oxidation behavior of the alloys.

All alloys were then aged at 1000 °C and then at 850 °C for 24 h respectively. The heat-treated cube was then cut into 1.7 mm thick slices using an IsoMet low speed saw by Buehler (Leinfelden-Echterdingen, Germany). Afterwards, the samples were ground with an 800 grid to ensure a homogeneous surface roughness.

Before the oxidation testing, the surface of the samples was measured via an outside micrometer and weighed in a Mettler Toledo AT20 (Mettler-Toledo International Inc., Greifensee, Switzerland). The samples were strung up by the whole in them in the furnace to minimize the contact area of the samples and guarantee a continuous oxidation (see Figure 1b). One sample of each alloy was heat-treated in the same furnace at 950 °C for 100 h, 300 h, and 500 h at air atmosphere and then air-cooled. The spallation products of the samples were not collected. Subsequently, the samples were weighed again in a Mettler Toledo AT20.

The samples were cold-embedded in Scandiplex by SCAN-DIA GmbH (Hagen, Germany) to avoid spallation of the oxide layer by the high pressure that is required by warm embedding. Afterwards, the samples were ground, polished, and finished with a vibration polishing OPS step. A TM3000 by Hitachi (Tokyo, Japan) was used for SEM imaging and EDX measurements. All images were made using a backscattered electron (BSE) detector.

## 3. Results

### 3.1. Classification of Oxidation Areas

**Figure 2:**
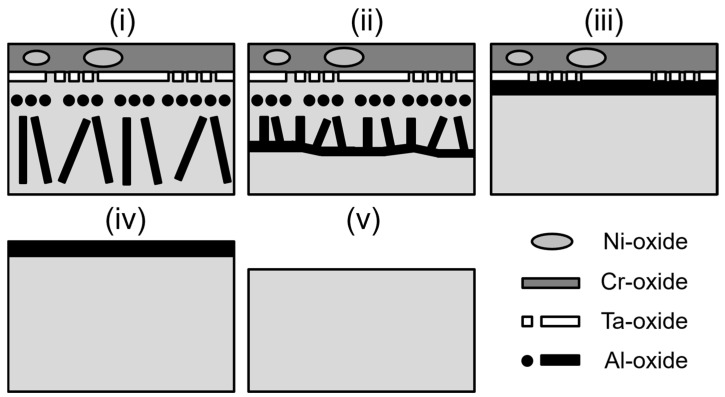
Schematic illustration of the different oxidation areas present in the SAM alloys.

The oxidation of SAM-Zr at 950 °C proceeds inhomogeneously. Therefore, not one single oxide layer formation mechanism can be given. However, there are recurring areas that can be found across the surface that can be distinguished. Those areas are schematically shown in Figure 2 in an idealized way. Area (i) consists of an outer Cr-oxide layer and a semi-continuous Ta-oxide layer underneath. The outer Cr-oxide layer can contain Ni-oxide islands, but does not in the case of SAM-1.9Hf. The region underneath those outer oxide layers is defined by internal Al oxidation in the form of individual particles. Area (ii) differs from area (i) only in the internal Al-oxide particles. They are not growing into the material indefinitely, but start to coalesce at a certain depth. Area (iii) differs from area (ii) by displaying a continuous Al-oxide layer directly underneath the semi-continuous Ta-oxide layer. In contrast to area (ii), there is no γ-matrix above the Al-oxide layer. Area (iv) is defined by a single continuous outer Al-oxide layer and area (v) is defined by a blank metal surface.

### 3.2. SAM-Zr

**Figure 3:**
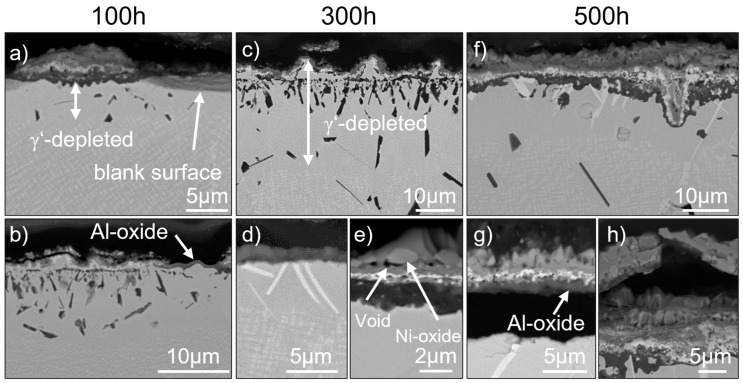
The surface of SAM-Zr after the exposure to 950 °C for 100 h (**a**,**b**), 300 h (**c**–**e**), and 500 h (**f**–**h**). Figure (**a**) shows area (i) (left) and (v) (right). Figure (**b**) shows area (i) (left) and area (iv) (right). Figure (**c**) shows area (i). Figure (**d**) shows area (v). Figure (**e**) shows area (iii). Figure (**f**) shows area (iii). Figures (**g**,**h**) show spallation of area (iii).

**Figure 4:**
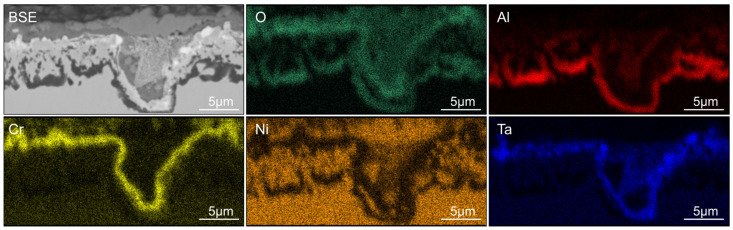
BSE image and EDX maps of O, Al, Cr, Ni, and Ta of the oxidation attack along a grain boundary of SAM-Zr after 100 h of oxidation at 950 °C. The figure shows a type (ii) area where the Al-oxide particles are in the process of growing together or have already grown together.

In SAM-Zr, area (i), like the other areas, is already present after 100 h of oxidation and is shown in Figure 3b,c. It consists of an outer Cr-oxide layer (dark gray) in which bright Ni-oxides (gray) can be found as well (see Figure 3e). Underneath this layer, a small semi-continuous Ta-oxide layer (bright gray) forms. Underneath the Ta-oxide layer, aluminum oxidizes internally (black elongated particles) and grows perpendicular to the surface into the material. Underneath this area, blocky and needle-like Al-nitrides form at some distance from the Al-oxide particles (see Figure 3c). Area (ii) can be observed after 300 h and 500 h. It is similar to area (i), but the Al-oxide particles coalesce and grow parallel to the surface (see Figure 4) instead of growing perpendicular to the surface (see Figure 3c). However, the horizontal growth of the Al-oxide particles occurs only rarely in SAM-Zr.

**Figure 5:**
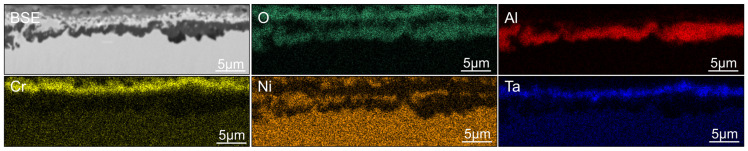
BSE image and EDX maps of O, Al, Cr, Ni, and Ta of a typical area (ii) of SAM-Zr after 100 h of oxidation at 950 °C. Most of the Al-oxide particles have already coalesced, but some particles on the left side remain separated.

Area (iii) is similar to area (i) and (ii), but instead of Al oxidizing internally, a continuous Al-oxide layer is formed underneath the Ta-oxide layer (see Figure 3e and Figure 5). Otherwise, the formation of the outer Cr-/Ni-oxide layer as well as the Ta-oxide layer is the same. Area (iv) occurs rarely and consists of a thin, single Al-oxide layer (see Figure 3b). This area is small and located between areas like (i), (ii), and (iii). Besides these areas, which show external oxide layers, area (v) only shows a blank surface without signs of oxidation or nitridation. Figure 3a shows this area (v) next to a small area (iii) after 100 h and Figure 3d shows area (v) after 300 h. Although the oxide layer has locally spalled off, features from underlying surfaces are still visible in the SEM images due to the three-dimensional nature of the sample and the two-dimensional cross-section. The formation of the η-phase, which can be seen in Figure 3d, is typical for area (v) in samples that were tested for 300 h and 500 h.

Generally, nitridation is not observed in every region. Still, it occurs more frequently in area (i) with Al-oxide particles that grow perpendicular to the surface instead of the areas (ii) and (iii), where an internal continuous Al-oxide layer forms.

The oxidation around the grain boundaries is also not homogeneous across the whole sample. In some areas, the oxidation behavior around a grain boundary that meets the surface does not change, but in other areas, the oxidation attack reaches deeper into the material (see Figure 4). The order of the oxide layers is similar to area (iii) and consists of an internal continuous Al-oxide layer around the grain boundary. This is followed by the γ-matrix, the Ta-oxide layer, and then the Cr-/Ni-oxide layer. The grain boundary itself consists largely of Ni-oxide and Ni-oxide mixed with Ta-oxides. No signs of oxidized carbides or other particles can be found at the grain boundary.

The blank surface of area (v) is a sign for spallation. This kind of spallation is the only one that can be observed after 100 h of oxidation. However, after 300 and 500 h of oxidation, spallation also occurs in areas (i), (ii), and (iii). Figure 3g and h show spalled areas, in which the spalled oxide is still partly attached to the sample. This shows that spallation occurs between the Cr-/Ni-oxide layer and the Ta-oxide layer (see Figure 3h) or between the continuous Al-oxide layer and the substrate (see Figure 3g). However, besides the detachment from the underlying oxide layer or substrate, the areas in which spallation occurs do not differ from the other areas.

Small voids appear already after 100 h of oxidation underneath the Ta-oxide layer and between the Cr-oxide and the Ni-oxide in the mixed Cr-/Ni-oxide layer (see Figure 3g).

SAM-Zr forms TaC [1], which dissolves during oxidation and forms a depleted zone. The γ’-phase dissolves as well, but the size of the depletion zone depends on the area. After 100 h, area (iii) with a continuous Al-oxide layer shows a small depletion zone of approx. 7 µm, which does not change with increasing duration. Area (v) with its blank surface shows the smallest depletion zone, which is hardly existent after 100 h (see Figure 3a). The depletion zone in area (i), exhibiting internal Al-oxide particles that grow perpendicular to the surface, is the largest.

In the γ′-depleted zone underneath the surface, plate- and needle-like particles form. EDX measurements have shown that they are enriched in Ta and depleted in Cr and Mo. Thermo-Calc calculations predict the formation of a Ta-rich η-phase at 950 °C, which is in good agreement with the morphology and composition of the observed particles. Therefore, this phase is assumed to be the Ta-rich η-phase. Some individual particles can be observed already after 100 h of oxidation and their number increases with increasing duration of the oxidation. It can be most frequently observed in areas where only an Al-oxide formed or the surface is blank without any oxide layer (see Figure 3d). After 500 h, it is also present in areas with a continuous Al-oxide layer underneath the Ta-oxide layer.

**Figure 6:**
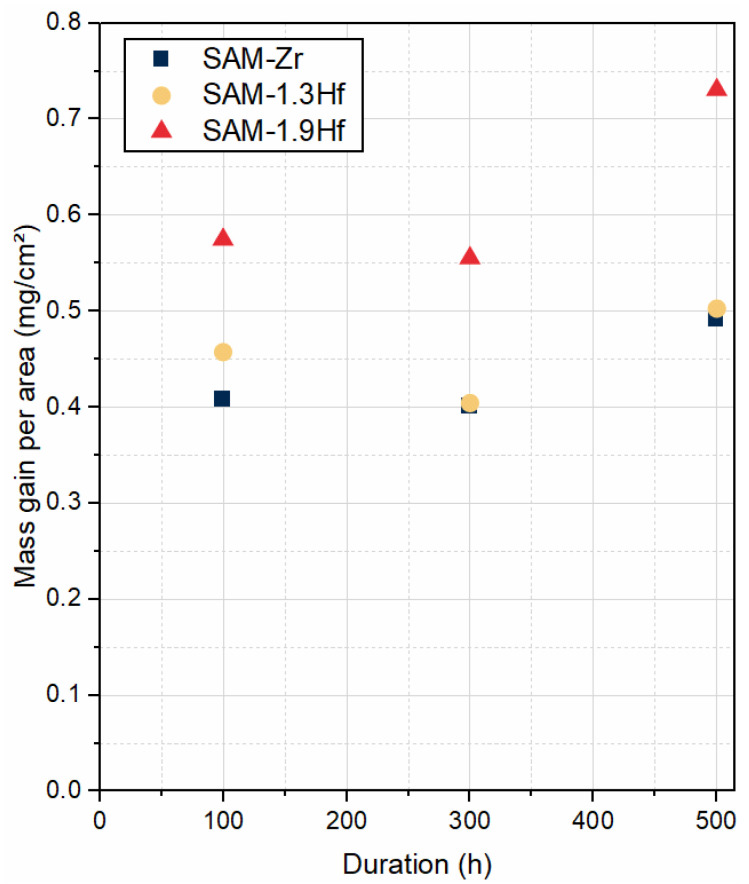
Mass gain per unit area measured after cooling the samples to room temperature. Spalled oxides were not taken into account.

Figure 6 shows the mass gain per area of the samples. The mass was weighed after the samples had cooled down from 950 °C, and the spalled oxides were not collected and are therefore not included in the measurement. Since indications of spallation can be seen in all samples of SAM-Zr, it has to be assumed that the real mass gain during oxidation is higher, and no information about the quantitative oxidation kinetics can be given. Nonetheless, even without the spalled oxides, the mass gain per area is positive.

### 3.3. SAM-1.3Hf

**Figure 7:**
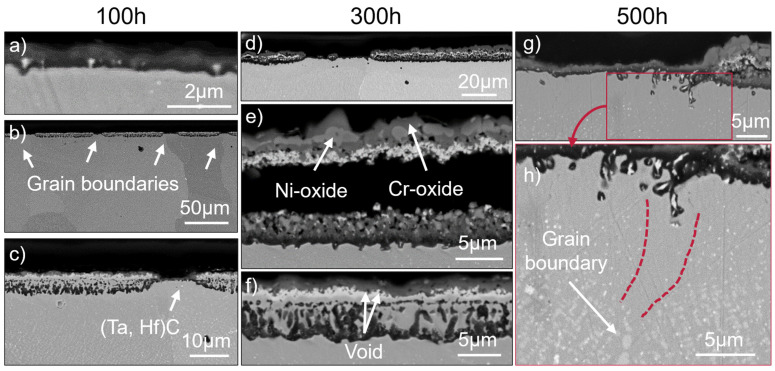
The surface of SAM-1.3Hf after the exposure to 950 °C for 100 h (**a**–**c**), 300 h (**d**–**f**), and 500 h (**g**,**h**). Figure (**a**) shows a detailed section of the Al-oxide layer of an area (iv). Figures (**b**–**d**) show area (iv) at grain boundaries and area (ii) between those grain boundaries. Figure (**e**) shows spallation of area (iii). Figure (**f**) shows area (ii). Figure (**g**) shows area (iv) on the left side and area (iii) on the right side. The red dashed lines in figure (**h**) mark the carbide depleted region around a grain boundary.

The oxidation behavior of SAM-1.3Hf differs from that of SAM-Zr, because it is more homogeneous. The surface is dominated by areas of type (ii) and (iii). Nonetheless, other areas can be distinguished as well. Area (i) can only be found after 100 h (see Figure 7c), because it evolves into area (ii) by the horizontal coalescence of internal oxidized Al particles after 300 h (see Figure 7f). Like in SAM-Zr, the outer Cr-oxide (dark gray) layer contains Ni-oxide (gray) as well (see Figure 7e). A Ta-oxide layer forms underneath it and then the internal oxidized Al follows. In contrast to SAM-Zr, no Al-oxide particles are present that grow as elongated individual particles perpendicular to the surface, because they stop growing into the material once they coalesce to an internal Al-oxide layer. The Al-oxide particles grow together much faster than in SAM-Zr and form an almost continuous internal Al-oxide layer after 100 h of oxidation (see Figure 7c). However, there are still areas in which this process has not finished and some spaces between the Al-oxide particles are still present. After 300 h, nearly all Al-oxide particles grow together to form an internal Al-oxide layer (see Figure 7f) and only a few type (i) areas are left. Eventually, after 500 h, there are no type (i) areas left in which the Al-oxide particles do not grow together.

Area (iii), with a continuous Al-oxide layer underneath the Ta-oxide layer, cannot be observed after 100 h of oxidation, but after 300 h and 500 h (see Figure 7d,e,). Area (iv), which is characterized by a single thin Al-oxide layer, is wider and can be found more frequently than in SAM-Zr (see Figure 7a–c). The alumina layer in Figure 7a appears dark in the SEM image and locally exhibits a very thin, slightly brighter contrast. Although this contrast suggests the presence of an additional oxide layer (e.g., Cr- or Ni-rich oxides), it may alternatively result from an SEM edge effect. Since EDX analyses show only Al enrichment and no detectable chromium or nickel enrichment, the presence of an additional oxide layer cannot be confirmed.

After 500 h of oxidation, the whole surface is either covered with a single outer Al-oxide layer (area iv) or an internal continuous Al-oxide layer (area ii and iii) (see Figure 7g,h).

In contrast to SAM-Zr, the areas around grain boundaries are type (iv) areas (see Figure 7b,c) and deep oxidation attacks with large Ni-oxide formation cannot be found. Similar to SAM-Zr, grain boundaries at which the oxidation behavior is unaffected can be observed as well. The only exception to the absence of deeper oxidation attacks along grain boundaries is one grain boundary at which a hot crack connected to the surface and enabled O to penetrate deeper into the material.

**Figure 8:**
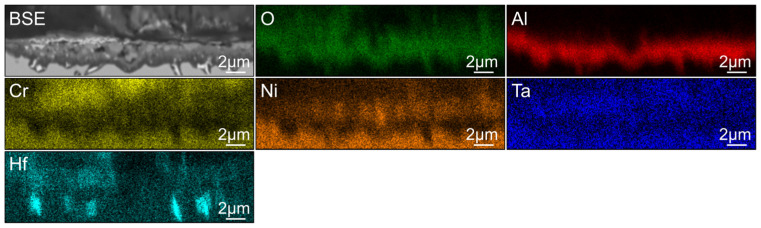
BSE image and EDX maps of O, Al, Cr, Ni, Ta, and Hf of the internal Al-oxide layer of SAM-1.3Hf after 500 h of oxidation at 950 °C. It shows area (iii) and the outer oxide layer spalled between the Ta-oxide and the γ-matrix.

Another significant difference between SAM-1.3Hf and SAM-Zr is the presence of Hf-oxide particles (bright particles) within the Al-oxide layer, which are already present after 100 h of oxidation and can best be seen in Figure 7a,g–h. After 100 h, the particles are equiaxed and with increasing duration of the oxidation, some particles coarsen and get elongated perpendicular to the surface. The Hf-oxide particles are always surrounded by Al-oxide, which leads to extrusions in the Al-oxide layers already after 100 h. These extrusions are so-called “pegs”, which can best be seen in Figure 7a,e,h and Figure 8.

The spallation of oxide layers can be observed as well in SAM-1.3Hf, but differs from that of SAM-Zr. In contrast to SAM-Zr, spallation does not occur after 100 h of oxidation, but after 300 and 500 h (see Figure 7e and Figure 8). The spallation occurs exclusively between the mixed outer Cr-/Ni-oxide layer and the underlying Ta-oxide layer. No signs of spalled Al-oxide layers can be found in any of the samples of SAM-1.3Hf. However, similar to SAM-Zr, voids can be observed underneath the Ta-oxide layer after 100 h of oxidation (shown here only after 300 h in Figure 7f) and after 300 h between the Cr-oxide and Ni-oxide in the mixed outer Cr-/Ni-oxide layer as well (see Figure 7e). Type (iv) areas, where a single thin Al-oxide layer is formed, show no void formation.

In contrast to the Hf free SAM-Zr, the η-phase is not present and the depletion zone of the γ′-phase in general is smaller (see Figure 7h). The depletion zone of the same areas is similar, but SAM-1.3Hf does not show area (i) with Al-oxide particles that grow perpendicular to the sample surface after 300 h and these areas show the largest γ′-phase depleted zones. Therefore, the γ′-depleted zone of SAM-1.3Hf is generally smaller than that of SAM-Zr.

Instead of TaC, SAM-1.3Hf forms (Ta, Hf)C [1] (see Figure 7c). This type of MC carbide is more stable and can still be observed right below the Al-oxide layer up to 300 h of oxidation. However, after 500 h, the (Ta, Hf)C close to the Al-oxide layer dissolves and a small depleted zone forms.

The mass gain per surface area of SAM-1.3Hf (see Figure 6) is positive. As mentioned above, the spalled oxides were not accounted for in the weight measurement after the cooling, but their inclusions would only increase the positive weight change.

### 3.4. SAM-1.9Hf

**Figure 9:**
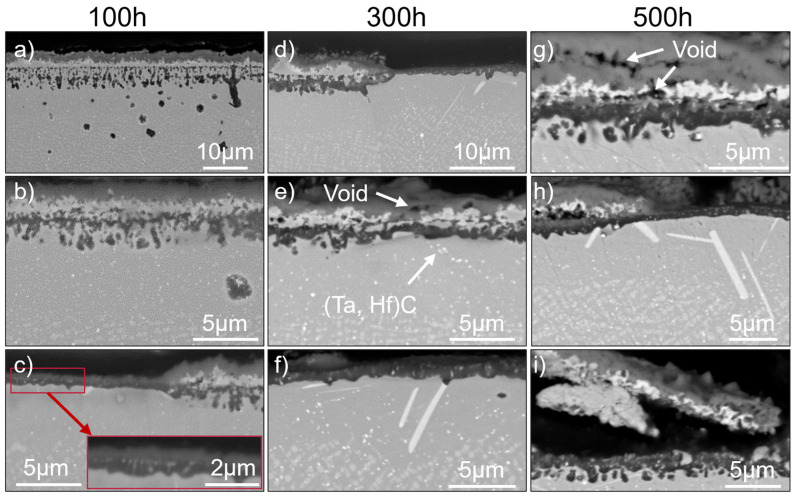
The surface of SAM-1.9Hf after the exposure to 950 °C for 100 h (**a**–**c**), 300 h (**d**–**f**), and 500 h (**g**–**i**). Figure (**a**) shows area (i). Figure (**b**) shows area (ii). Figure (**c**) shows area (iv) on the left and (i) on the right side. Figure (**d**) shows area (ii) on the left and area (iv) on the right side. Figure (**e**) shows area (iii). Figure (**f**) shows area (iv). Figure (**g**) shows area (iii). Figure (**h**) shows area (iii) on the left and area (iv) on the right side. Figure (**i**) shows spallation of area (iii).

The oxidation behavior of SAM-1.9Hf is very similar to that of SAM-1.3Hf and differs only slightly. One of those differences is that SAM-1.9Hf does not form an outer Cr-/Ni-oxide layer in the areas (i–iii) like SAM-Zr and -1.3Hf, but a Cr-oxide layer (see Figure 9g). No Ni-oxide islands can be observed. However, these areas are otherwise identical to the areas that form a Cr-/Ni-oxide layer in SAM-Zr and -1.3Hf.

After 100 h of oxidation, areas of type (i), which consist of an outer Cr-oxide layer, an underlying Ta-oxide layer, and internal oxidized Al, can be seen (see Figure 9a). Additionally, areas of type (ii), which are similar to type (i), but consist of coalesced internal Al-oxide particles instead of individual ones (see Figure 9b), can be observed. After 300 h, this process is completed in almost all former areas of type (i) and only type (ii) areas are left (see Figure 9d).

Similar to SAM-1.3Hf, areas of type (iii) with a continuous Al-oxide layer below the outer Cr- and Ta-oxide layer (see Figure 9e,h), as well as areas of type (iv) with a single outer Al-oxide layer (see Figure 9c,d,f), can be observed after all durations. As in SAM-1.3Hf, a contrast is observed within the alumina layer. However, EDX analyses show only aluminum enrichment and no evidence of an additional oxide layer.

The increased Hf content in SAM-1.9Hf does not change the appearance of the Hf-oxide particles. Similar to SAM-1.3Hf, they are already present after 100 h of oxidation as fine and equiaxed particles surrounded by Al-oxide (see Figure 9c). Some of them coarsen and grow elongated into the material, but others remain as fine particles even after 500 h (see Figure 9g).

The grain boundaries of SAM-1.9Hf exhibit no deep oxidation attack with Ni-oxide formation, as observed in SAM-Zr, but instead behave similarly to those of SAM-1.3Hf. The oxidation behavior is either unaffected or type (iv) areas with a single outer Al-oxide layer are present around them. However, some grain boundaries display coarse, elongated Al-oxide particles along the boundary, which penetrate deeper into the material than the surrounding Al-oxide particles formed within the grain interior. (see Figure 9a).

Spallation can also be observed in SAM-1.9Hf, but occurs less frequently and between the Ta-oxide and Al-layer (see Figure 9i). The Al-oxide layer shows good adhesion and no indications of its spallation can be found.

The γ’-depleted zone below the oxide layers is also similar to SAM-1.3Hf and, in general, smaller than that of SAM-Zr. SAM-1.9Hf also forms (Ta, Hf)C MC-carbides that remain stable close to the oxide layer, which can best be seen in Figure 9e,f (small bright particles).

In contrast to SAM-1.3Hf, SAM-1.9Hf shows the formation of the Ta-based η-phase. The morphology of this phase is plate- and needle-like and it forms in the γ′-depleted zone. The η-phase is not present after 100 h of oxidation, but can be found after 300 and 500 h and it forms preferably in type (iv) areas, where a single outer Al-oxide layer forms during oxidation (see Figure 9d,f,h). Type (i), (ii), and (iii) areas with Cr-oxide and Ta-oxide layers do not show the presence of η-phase after 300 h, but after 500 h, if they are next to a type (iv) area.

Similar to both other alloys, voids are present after all durations and become more frequent over time. They form underneath the Ta-oxide layer (see Figure 9e,g) and in the Cr-oxide layer. No voids are present at the Al-oxide layer, and therefore, no voids are present in type (iv) areas.

The weight change of SAM-1.9Hf after the oxidation tests is positive (see Figure 6).

## 4. Discussion

### 4.1. Oxidation Behavior

Considering the Al content of 3 wt.% and the Cr content of 14 wt.%, the alloys would be considered as typical Cr-oxide-forming alloys of type II [4,23]. In general, an Al content of 4.5 wt.% is considered necessary to change the dominating oxide mechanisms from an outer Cr-oxide layer to an outer Al-oxide layer [24]. However, at 950 °C, all three alloys exhibit areas where a single continuous outer Al-oxide layer can be found, which indicates that the composition of the SAM alloys is on the threshold between type II and type III. This is likely caused by the high Ta content, which is reported to promote the formation of an Al-oxide layer [9]. It is even further promoted by the addition of Hf in SAM-1.3Hf and -1.9Hf, which show an outer Al-oxide layer more frequently than SAM-Zr.

However, in all three alloys, the majority of the surface is protected by either an outer Cr-/Ni-oxide or a Cr-oxide layer. SAM-Zr and -1.3Hf exhibit no continuous Ni-oxide layer but small particles of Ni-oxide inside the Cr-oxide layer (see Figure 3e and Figure 7e). These particles are too small and the oxide layers too thin for an accurate quantitative EDX analysis. A phase analysis via XRD (X-ray diffraction) might give more valuable information but is beyond the scope of the current work. This mixed oxide layer is less protective than a single Cr-oxide layer and tends to spall more than a pure Cr_2_O_3_ layer [25]. In contrast, SAM-1.9Hf does not show Ni-oxides inside the outer Cr-oxide layer (see Figure 9g). The high amount of Hf seems to promote the formation of a Cr-oxide layer before Ni-oxides can form. This is in good agreement with the oxidation of grain boundaries, which show Ni-oxide in SAM-Zr, but not in -1.3Hf and -1.9Hf.

Areas where an outer Cr-/Ni-oxide or Cr-oxide forms also exhibit a Ta-rich oxide layer underneath the Cr-/Ni-mixed oxide layer (see Figure 4, Figure 5, Figure 7f and Figure 9e,g). This layer is characteristic of Ta-containing Ni-based superalloys and is often reported elsewhere [8,9,10]. The EDX maps show that the layer with extensive Ta enrichment is also depleted in Ni and Cr, which indicates a Ta-oxide layer. Unlike the outer Cr-/Ni-mixed oxide layer, the Ta-oxide layer is not affected by the addition of Hf.

However, the addition of Hf affects the formation of an outer Al-oxide layer and the internal Al oxidation underneath the above-mentioned layers. The Al-oxide particles grow not as deeply into the material during the internal oxidation as in SAM-Zr (compare Figure 3b,c with Figure 7c,f and Figure 9a,b). Additionally, the Al-oxide particles grow together horizontally to form an internal Al-oxide layer (see Figure 7c,f and Figure 9d). This is more beneficial than the Al-oxide particles growing perpendicular to the surface in SAM-Zr (see Figure 3c), because the continuous internal layer hinders further oxidation. Type (iii) areas that show a continuous Al-oxide layer beneath the Ta-oxide layer are also more frequent in the Hf-containing alloys and are more common after 300 h and 500 h. However, it is not clear if those areas formed a continuous Al-oxide layer initially or if the Al oxidized internally and grew together horizontally and only appears as a continuous layer afterwards. The increased occurrence of type (iii) areas after 300 h and 500 h suggests that they evolve from type (ii) areas, because it takes some time for the metal above the internal Al-oxide layer to oxidize. Nonetheless, the addition of Hf results in a better oxidation resistance of SAM-1.3Hf and -1.9Hf, because they are either protected by

- A single, continuous, outer Al-oxide layer;
- An internal continuous Al-oxide layer that is formed by the coalescence of internal oxidized Al;
- A continuous Al-oxide layer that formed initially underneath the Ta-oxide layer.

The reason as to why the Al-oxide particles of SAM-1.3Hf and -1.9Hf coalesce internally instead of growing deeper into the material remains unknown, but assumptions can be made. One explanation might be that the nucleation density of internal Al-oxide particles is higher, which results in a tighter spacing of the particles, which promotes the coalescence. The shortest distance between two Al-oxide particles is approx. 1.53 ± 0.83 µm in SAM-Zr, 0.60 ± 0.26 µm in SAM-1.3Hf, and 0.59 ± 0.22 µm in SAM-1.9Hf. The higher nucleation density might be caused by Hf-oxide particles that form early during the oxidation process or by (Ta, Hf)C particles that are finer and more stable than TaC, which is already dissolved after 100 h of oxidation (compare Figure 3c with Figure 7c and Figure 9c). Additionally, fine and bright particles can be found in the coalesced Al-oxide particles as well (see Figure 7f). These particles are likely also either (Ta, Hf)C or Hf-oxide particles. If Al-oxide nucleates at those particles between two Al-oxide rods that grow into the material, it will favor their coalescence.

This explanation is in good agreement with Wagner’s criterion for the transition from internal to external oxidation. Wagner [26] proposed that this transition occurs when the volume fraction of internally precipitated oxide particles becomes sufficiently large that diffusion paths within the metallic matrix are significantly constricted. Since oxygen and metal ions do not diffuse through the oxide particles themselves, diffusion must occur around them through the surrounding matrix. As the oxide volume fraction increases, the effective metallic diffusion cross-section decreases, which promotes particle coalescence and ultimately leads to the formation of a continuous external oxide layer.

A higher nucleation density of Al-oxide particles, and thus a smaller particle spacing, would locally increase the oxide volume fraction and further reduce the available diffusion cross-section. This may promote early particle impingement and could therefore explain the horizontal coalescence of the internal Al-oxide particles observed in the Hf-containing alloys.

It is not possible to quantitatively assess the oxidation resistance of the alloys in this study by the weight change, because the cross-sections show spallation of the oxide layers during cooling that are not accounted for by the measurement. Therefore, only a qualitative assessment based on the cross-sections can be made. From that assessment, it can be drawn that the oxidation resistance of SAM-Zr is sufficient during isothermal conditions, but it will very likely suffer catastrophic oxidation due to its poor oxide layer adhesion. The addition of Hf in SAM-1.3Hf and -1.9Hf improves their oxidation resistance by promoting the Al-oxide layer formation and its adhesion. The spallation of the Cr-/Ni-oxide layer does not pose as big a threat as it does for SAM-Zr, because a continuous Al-oxide layer forms underneath. Cyclic oxidation tests to confirm these assumptions are going to be part of future work.

### 4.2. Grain Boundary Oxidation

It can be observed that the oxidation behavior of the alloys behaves differently where grain boundaries meet the surface. This is most obvious in SAM-Zr, which does not contain Hf. The oxidation attack reaches deeper into the material along the grain boundaries (see Figure 4), which indicates that O penetrates faster into the material along the grain boundaries than the metal ions diffuse outwards. The addition of Hf eliminates these deep oxidation attacks along grain boundaries. Instead, areas with a single continuous outer Al-oxide layer can be found around grain boundaries (see Figure 7b,c and Figure 9d). One reason for this phenomenon could be that either the diffusion of Al along the grain boundary is increased or the diffusion of O is decreased. An increase in the Al diffusion velocity would be contrary to the findings of other studies, where the addition of Hf leads to a decreased growth rate of the Al_2_O_3_ layer, which is believed to be caused by a reduction in the outwards diffusion of Al cations [15,22]. Another explanation, which is in better agreement with other studies, is the formation of Hf-oxides. Of all the alloying elements, Hf has the highest affinity to oxygen. This can also be observed in the as-printed microstructure, where SAM-Zr shows a few Al-oxide particles that either formed during the PBF-LB/M process or were already present in the powder. In contrast, the Hf containing alloys show Hf-oxide particles instead. Hf does not form an oxide layer at the surface, but small Hf-oxide particles that are reported to promote the formation of Al_2_O_3_, because they can act as nucleation sites [17]. The early formation of these oxide particles binds O at the surface in the beginning phase of the oxidation until either an Al-oxide or a combination of a Ni-/Cr-oxide and Ta-oxide layer forms that prohibits further oxidation. Hf is also a grain boundary active element, and it was shown that it segregates towards grain boundaries in SAM alloys [1]. More Hf-oxide particles at grain boundaries could favor the nucleation of Al-oxide and thus explain why the Hf-containing alloys often show a single continuous outer Al-oxide layer around grain boundaries.

### 4.3. Oxide Layer Spallation

The spallation of oxide layers can be observed in the cross-section of all alloys. Unfortunately, the tests were conducted in a furnace that did not allow for monitoring the weight change during the oxidation test. However, the weight change before and after the test is positive. Also, no signs of excessive nitridation or other changes of the microstructure can be found that would indicate an oxide layer spallation during high-temperature exposure. Therefore, it can be assumed that the spallation took place during the subsequent air cooling.

SAM-Zr differs from SAM-1.3Hf and -1.9Hf by showing blank metal surfaces (see Figure 3a,d) and spallation between the Al-oxide layer and the metal surface (see Figure 3g). Unfortunately, no connected oxide layer can be found in the vicinity of the blank metal surfaces. However, there are indications that these areas were once protected by an Al-oxide layer. One indication is that this area shows no nitrides underneath it. An Al-oxide layer is an effective barrier against N, whereas Cr-oxide layers can transmit N [27]. This indicates that an Al-oxide layer had formed before nitrides could form. Another indication is the presence of the η-phase. This phase can be most frequently found in areas where an Al-oxide layer has formed (see Figure 9f,h), because the depletion of Al favors the formation of the η-phase. Also, no internal Al-oxidation took place, which only happens in regions where an Al-oxide layer formed. Therefore, it is assumed that the Al-oxide layer of SAM-Zr has a weak adhesion, which will be a problem during cyclic oxidation conditions.

Ta is reported to reduce the stability of the protective Al-oxide layer by forming Ta_2_O_5_ [9], but this can neither be denied nor confirmed in this study, because no remains of the spalled oxidation layer were found. The Al-oxide layer is generally known to display a weak adhesion, and since no elements are added that would improve its adhesion, this might be the simple explanation for its spallation. SAM-Zr does contain Zr, which is also known to improve the oxidation resistance, but compared to other alloys, its content of 0.02 wt.% is low and insufficient. Most often, elements like Hf and/or Y are alloyed to increase the adhesion of the Al-oxide layer [28]. The positive effect of Hf can be seen in SAM-1.3Hf and -1.9Hf, because neither of those alloys shows the spallation of the Al-oxide layer. This can be linked to the formation of Hf-oxide particles that nucleate Al-oxide around them (see Figure 7e,c), which is also mentioned in other studies [17,18,19,21]. The thickness of the Al-oxide layer is larger where Hf-oxide particles are present. This leads to a rough Al-oxide/metal interface after 100 h, which increases the adhesion. After 500 h, the coarsening of the Hf-oxide particles leads to pronounced pegs that grow into the material perpendicular to the surface (see Figure 7g,h and Figure 9g), which increases the adhesion even more. In contrast, in the few cases where the Al-oxide layer can be observed in SAM-Zr, its Al-oxide/metal interface is smooth (see Figure 3b).

SAM-Zr and -1.3Hf show also spallation of the Cr-/Ni-oxide layer, which is known to exhibit a higher risk of spallation than a Cr-oxide layer [25]. In this case, the identification of the phases that are involved and the location where the spallation begins is easier, because, often, the spalled Cr-/Ni-oxide layer is still partly attached to the sample. It can be seen that the spallation occurs between the Cr-/Ni-oxide layer and the Ta-oxide layer (see Figure 3h and Figure 7e). The spallation might be caused by the formation of voids that form at the interfaces between different oxides (see Figure 3e and Figure 7f). They increase in number and size with increasing duration. A different thermal expansion of the Ta-oxide and the Cr-/Ni-oxide leads to stress during cooling, which, coupled with the weakening of the interface by voids, then leads to the spallation of the Cr-/Ni-oxide layer. However, this cannot be the sole reason for the spallation between the Cr-/Ni-oxide layer and the Ta-oxide layer, because SAM-1.9Hf shows these voids as well (see Figure 9g) and the spallation of this alloy occurs between the Ta-oxide and the Al-oxide layer. This indicates that the adhesion of the Cr-/Ni-oxide layer is weaker than that of the Cr-oxide layer. The adhesion of the Ta-oxide layer to the Al-oxide layer is weak as well, which is why spallation still occurs in SAM-1.9Hf. However, the spallation between Ta- and Al-oxide in SAM-1.9Hf occurs not as frequently as that between the Cr-/Ni- and the Ta-oxide in SAM-Zr and -1.3Hf.

In general, the oxide layer spallation poses a bigger threat on SAM-Zr, because the Hf-containing alloys form a continuous protecting Al-oxide layer underneath, which offers further protection, even if the Cr-/Ni- or Cr- and Ta-oxide spalls.

### 4.4. Microstructure Underneath the Oxide Layer

The formation of an oxide layer during high-temperature exposure also causes the microstructure in the vicinity of the surface to change. The regions underneath the oxide layer act as a reservoir for elements needed to form the oxide layer. Therefore, this region is depleted in Al and Ta if an Al-oxide or Ta-oxide layer forms. This results in the dissolution of the γ′-phase and creates a γ′-phase-depleted zone, which can be seen in all alloys. The size of these γ′-phase-free zones differs between different areas in one alloy. Areas, where a Ta-oxide layer forms and Al oxidizes internally, show the largest γ′-phase-depleted zones (see Figure 3c), because, there, the most γ′-phase-forming elements are consumed. Whereas areas, where a single continuous outer Al-oxide layer forms, show the smallest γ′-phase-depleted zone (see Figure 3d), because once the small layer develops, further oxidation and consumption of γ′-phase-forming elements is strongly retarded. In total, the γ′-phase-depleted zone of SAM-1.3Hf and -1.9Hf is smaller since the regions that are covered by Al-oxide are more frequent.

Besides the γ′-phase-depleted zone, a carbide-depleted zone can be observed. Both alloys contain MC carbides after the aging heat treatments: TaC in SAM-Zr and (Ta, Hf)C in SAM-1.3Hf and -1.9Hf. The TaC-depleted zone in SAM-Zr is larger than the γ′-phase-depleted zone, even after 100 h, and the size of the zone increases with increasing duration. In contrast, SAM-1.3Hf and -1.9Hf show no visible carbide-depleted zone after 100 h and 300 h (see Figure 7a and Figure 9e). Only after 500 h can a small carbide-depleted zone be seen, which is still smaller than the γ′-phase-depleted zone (see Figure 7h and Figure 9h). This carbide-depleted zone is likely caused by the coarsening of the Hf-oxide particles that consume Hf. The higher stability of (Ta, Hf)C over TaC during oxidation is in good agreement with the higher thermal stability of (Ta, Hf)C that was already reported in a different study [1]. The high stability of the (Ta, Hf)C and its fine particle size might be the reason why overdoping effects of Hf, that are reported in other studies, cannot be seen in PBF-LB/M-processed SAM-1.3Hf and -1.9Hf. Some of these overdoping effects are connected to the dissolution of MC carbides and the subsequent changes in the microstructure or the oxidation of coarse MC carbides at the surface. In general, the addition of 1.3 wt.% and 1.9 wt.% Hf can be considered beneficial, because it keeps the depleted zones smaller, which otherwise would deteriorate the mechanical properties.

Another difference between the alloys is the presence of the detrimental Ta-rich η-phase in surface-near regions. The η-phase only forms in SAM-Zr and SAM-1.9Hf and a significant number of particles can be observed after 300 h (see Figure 3d and Figure 9f). In contrast, SAM-1.3Hf shows no η-phase formation. Whether the η-phase forms depends on the (Ta+Hf+Ti+Nb)/Al ratio [29]. The high Ta and moderate Al content of the SAM alloys already puts these alloys at a high risk of η-phase formation. However, after 500 h at 950 °C in this study and more than 3000 h at 850 °C in another study [1], no signs of η-phase formation inside the material can be found. Therefore, it can be concluded that the formation of the η-phase underneath the surface is caused by local deviations from the bulk composition that promote the formation of the η-phase.

The formation of oxide layers causes such local deviations. The formation of Al-oxide increases the risk of η-phase formation, while the formation of a Ta-oxide layer decreases it. The absence of the η-phase in SAM-1.3Hf cannot be explained by the formation of different oxide layers, because SAM-1.3Hf and -1.9Hf are similar. However, there is an explanation that involves two contrary mechanisms.

On the one hand, the addition of Hf reduces Al consumption by improving oxidation resistance, which is beneficial as discussed above. On the other hand, Hf addition shifts the (Ta+Hf+Ti+Nb)/Al ratio in the matrix toward less favorable values. When increasing the Hf content from 1.3 wt.% Hf to 1.9 wt.%, no further improvement in oxidation resistance is observed, while the (Ta+Hf+Ti+Nb)/Al ratio continues to shift unfavorably. As a consequence, η-phase formation reoccurs at 1.9 wt.% Hf. These two opposing mechanisms result in an optimal compositional window of around 1.3 wt.% Hf with respect to stability against η-phase formation in the vicinity of the oxide layer.

## 5. Conclusions

The isothermal oxidation behavior of three alloys with different Hf contents exposed at 950 °C for 100 h, 300 h, and 500 h was qualitatively examined in this work by cross-sections and the measurement of the weight change.

The addition of 1.3 wt.% and 1.9 wt.% Hf shows a beneficial effect on oxidation resistance, as the alloys are protected by an Al-oxide layer, despite their low Al content. This finding is particularly interesting for PBF-LB/M-processed alloys, where a high Al content can lead to strain-age cracking. More demanding cyclic oxidation tests and quantitative studies are planned as future work. The new Ni-based superalloy without Hf addition shows a weak adhesion of the Al-oxide and the Cr-/Ni-oxide layer that leads to the spallation of those layers during cooling.

The following conclusions can be drawn from this study:

(1) The new Ni-based superalloy without Hf addition exhibits weak adhesion of both the Al-oxide and Cr-/Ni-oxide layers, which leads to spallation of these layers during cooling.
(2) The addition of Hf promotes the formation of an Al-oxide layer and improves its adhesion in SAM-1.3Hf and SAM-1.9Hf, thereby increasing oxidation resistance. A continuous Al-oxide layer forms either externally or internally through the coalescence of internally oxidized Al-oxide particles.
(3) The addition of 1.3 wt.% Hf does not improve the adhesion of the Cr-/Ni-oxide layer to the Ta-oxide layer. However, increasing the Hf content to 1.9 wt.% leads to a transition from a Cr-/Ni-oxide layer to a Cr-oxide layer, which exhibits better adhesion to the Ta-oxide layer.
(4) η-phase formation occurs both without Hf and at 1.9 wt.% Hf due to opposing effects of Hf on oxidation resistance and matrix chemistry, resulting in an optimal stability window against η formation at intermediate Hf contents of approx. 1.3%.

## 6. Patents

The compositions of the three investigated alloys are covered under the patent “Nickel-Base Alloy Composition for Component Parts with Reduced Susceptibility to Cracking and Optimized High-Temperature Properties” (US20240011128, filed 22 September 2023) [3]. All experimental work reported in this study was conducted in compliance with the applicable intellectual property regulations associated with this patent.

## Figures and Tables

**Table 1:** Chemical composition of SAM-Zr, -1.3Hf, and -1.9Hf in wt.% obtained via wet chemistry analysis.

Alloy	Ni	Co	Al	Cr	Ta	Hf	Mo	W	C	B	Zr	S
SAM-Zr	Bal.	19.1	2.9	13.8	9.1	/	2.5	2.4	0.044	0.006	0.022	0.002
SAM-1.3Hf	Bal.	18.8	3.0	13.9	8.9	1.3	2.5	2.3	0.05	0.009	0.025	0.002
SAM-1.9Hf	Bal.	18.8	3.0	14.1	9.0	1.9	2.6	2.9	0.05	0.008	0.034	0.003

## Data Availability

The complete dataset cannot be made publicly available because the data are subject to confidentiality obligations and were generated in the context of an industrial collaboration. Further inquiries can be directed to the corresponding author.

## Abbreviations

The following abbreviations are used in this manuscript:

BSE	Backscattered electron
EDX	Energy-dispersive X-ray spectroscopy
OPS	Oxide polishing suspension
PBF-LB/M	Powder bed fusion by laser beam of metals
SAC	Strain-age cracking
SAM	Superalloy for additive manufacturing
SEM	Scanning electron microscopy
XRD	X-ray diffraction
